# Conformational change of Sos-derived proline-rich peptide upon binding Grb2 N-terminal SH3 domain probed by NMR

**DOI:** 10.1038/srep02913

**Published:** 2013-10-09

**Authors:** Kenji Ogura, Hideyasu Okamura

**Affiliations:** 1 Department of Structural Biology, Faculty of Advanced Life Science, Hokkaido University, Kita 21 Nishi 11, Kita-ku, Sapporo 001-0021, Japan; 2 Laboratory for Biomolecular Structure and Dynamics, Cell Dynamics Research Core, RIKEN Quantitative Biology Center, 1-7-22 Suehiro-cho, Tsurumi-ku, Yokohama, Kanagawa 230-0045, Japan

## Abstract

Growth factor receptor-bound protein 2 (Grb2) is a small adapter protein composed of a single SH2 domain flanked by two SH3 domains. The N-terminal SH3 (nSH3) domain of Grb2 binds a proline-rich region present in the guanine nucleotide releasing factor, son of sevenless (Sos). Using NMR relaxation dispersion and chemical shift analysis methods, we investigated the conformational change of the Sos-derived proline-rich peptide during the transition between the free and Grb2 nSH3-bound states. The chemical shift analysis revealed that the peptide does not present a fully random conformation but has a relatively rigid structure. The relaxation dispersion analysis detected conformational exchange of several residues of the peptide upon binding to Grb2 nSH3.

Src homology 3 (SH3) domains are small protein modules consisting of approximately 60 amino acids that occur in cytoplasmic proteins, tyrosine kinases, and cytoskeletal proteins known to play important roles in signal transduction pathways^1^. SH3 domains mediate protein-protein interactions by recognizing proline-rich peptide sequences within target proteins^2^. Many structures of SH3 domains complexed with their specific ligand peptides have been solved by X-ray crystallography and NMR spectroscopy. These structural studies have revealed that proline-rich peptides adopt a polyproline type II (PPII) helix on the SH3 domain^3^,^4^,^5^,^6^,^7^.

Growth factor receptor-bound protein 2 (Grb2) is a small adapter protein composed of a single SH2 domain flanked by two SH3 domains^8^. It has been shown that the N-terminal SH3 (nSH3) domain of Grb2 binds a proline-rich region present in the guanine nucleotide releasing factor, son of sevenless (Sos). The Grb2/Sos complex is an important component of a highly conserved pathway that transmits signals from the receptor to the nucleus and controls cell multiplication and differentiation^9^. Several structural studies for Grb2 nSH3 complexed with the Sos-derived peptide, which has a sequence of VPPPVPPRRR (denoted as VPP peptide), have been reported^10^,^11^,^12^. These structural studies have revealed the details of the interaction between the VPP peptide and the nSH3 domain. Grb2 nSH3 has three binding sites for the ligand peptide, denoted as S1, S2, and S3. The hydrophobic S1 and S2 sites bind Pro2-Pro3 and Val5-Pro6 of the VPP peptide, respectively. The negatively charged S3 site, which is crucial for the orientation of the bound peptide, binds the side chain of Arg8. Thus, the VPP peptide adopts a PPII helix and is bound to nSH3 in a class-II (minus) orientation with one face of the trigonal prism^13^.

The structure of the VPP peptide in the free state has been also investigated using NMR spectroscopy and circular dichroism^14^; the free VPP peptide shows a mixed conformer structure as a result of *cis*-*trans* isomerization around the Xaa-Pro bonds. Moreover, the VPP peptide is not a random coil; it takes on a ~60% PPII helix structure even in the free state.

As mentioned above, the conformation of the VPP peptide has been well investigated in both states, bound and free. However, conformational exchange of the VPP peptide upon binding Grb2 nSH3 remains unknown. Accordingly, we present here a ^13^Cα NMR relaxation dispersion analysis of the VPP peptide in the transition between free and bound states. NMR relaxation dispersion techniques are powerful tools for studying conformational dynamics or structural changes of biomolecules on the time scale of microseconds to milliseconds. Although isotope-enriched samples are commonly used for relaxation dispersion measurements, Peng et al. have demonstrated ^13^C relaxation dispersion at natural abundance levels for ligand peptides during protein binding^15^,^16^, which has thus provided a comprehensive analysis of μs-ms conformational dynamics related to binding of the ligands.

In this report, we first establish the sequential assignment of the ^13^C/^15^N-labeled VPP peptide in the free and Grb2 nSH3-bound states using triple resonance NMR experiments. We then analyse the conformational properties of the VPP peptide based on the chemical shifts of the main chain atoms for ^1^H, ^13^C, and ^15^N. Finally, we measure ^13^Cα relaxation dispersion for the VPP peptide at a natural abundance. Our NMR relaxation dispersion analysis of the VPP peptide upon binding Grb2 nSH3 reveals conformational exchange at specific residues of the peptide.

## Results

### Chemical shift-based conformational analysis for VPP peptide

**Figure 1:**
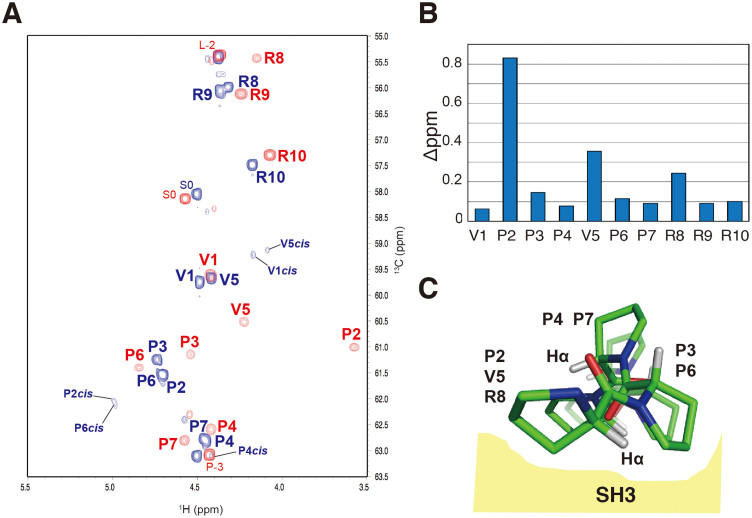
(A) ^1^H-^13^C HSQC spectra of ^13^C/ ^15^N-labeled VPP peptide. Free (blue) and Grb2 nSH3-bound (red) peptide spectra are overlaid. Cross-peaks are labelled with one-letter amino acid codes and the residue number. The minor peaks derived from the *cis* Xaa–Pro bond are also labelled. (B) Chemical shift differences between the free and Grb2 nSH3-bound states plotted as functions of amino acid sequence for the VPP peptide, where Δppm is defined as (0.5*[0.3*(Δω_C_)^2^ + (Δω_H_)^2^])^1/2^. (C) Schematic model of the binding of the VPP peptide to Grb2 nSH3.

We completed the resonance assignment of main chain atoms (^1^Hα, ^13^Cα, ^13^C′, and ^15^N) for the ^13^C/^15^N-labeled VPP peptide in the free and Grb2 nSH3-bound states. Because the VPP peptide contains five prolines among its ten residues, we conducted NMR experiments for the main chain assignment of proteins dissolved in ^2^H_2_O solution^17^. The chemical shifts of ^1^Hα, ^13^Cα, ^13^C′ and ^15^N for free and Grb2 nSH3-bound VPP peptide are shown in Table 1. Figure 1a shows the ^1^H-^13^Cα HSQC spectra of free and Grb2 nSH3-bound VPP peptide. Minor peaks derived from *cis* Xaa–Pro bonds were observed for residues of Val1, Pro2, Pro4, Val5, and Pro6 in the free state but not in the bound state. The relative intensities of the minor peaks with respect to the major peaks for these residues were 10% for Val1, 3% for Pro2, 14% for Pro4, 10% for Val5, and 6% for Pro6. These values showed good agreement with a previous report showing 9% minor (*cis*) conformation^14^. In contrast, the lack of minor peaks in the bound state indicates that the VPP peptide forms the PPII-helix on the surface of Grb2 nSH3.

The chemical shift changes of the ^13^C/^15^N labelled VPP peptide upon binding to unlabelled Grb2 nSH3 were investigated using the ^1^Hα-^13^Cα correlated peaks from the HSQC spectra (Figure 1a). Figure 1b shows the normalized chemical shift differences of ^1^Hα-^13^Cα pairs between the free and bound states, where ^13^Cα chemical shifts are scaled by a factor of 0.3^18^. Interestingly, Pro2, Val5, and Arg8 showed relatively large chemical shift changes upon binding to Grb2 nSH3 that may be explained by the schematic binding model of the PPII-helix on the SH3 domain (Figure 1c). As mentioned previously, when proline-rich peptides bind to SH3 domains, the peptides form a PPII-helix and contact the SH3 domains using one face of the trigonal prism. In the case of the VPP peptide, Pro2–Pro3 and Val5–Pro6 correspond to the basal planes of the trigonal prism, where Hα of both Pro2 and Val5 is deeply stuck into the binding sites of the SH3 domain as shown in Figure 1c. In particular, Pro2 is surrounded by aromatic residues: Tyr7 and Tyr52 of Grb2 nSH3. Similarly, Val5 is bound in the pocket formed by Phe9 and Trp36. Therefore, Pro2 and Val5 are extremely perturbed by the ring-current shift of aromatic side chains upon Grb2 nSH3-binding. The ^13^Cα-perturbation of Pro2 was less extreme than that of ^1^Hα likely because of the lack of changes in the dihedral angles around this residue. Totally, the chemical shift perturbation analysis is in good agreement with the structural studies based on NMR spectroscopy^10^,^11^,^12^.

**Figure 2:**
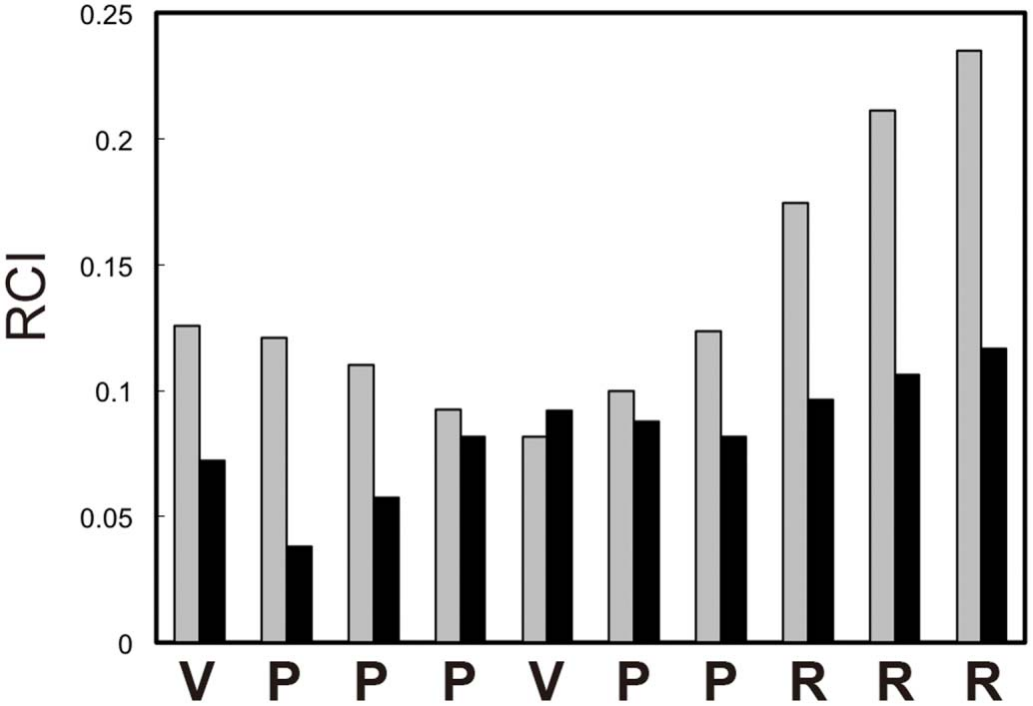
Random Coil Index of the VPP peptide in the free state (grey) and the bound state for Grb2 nSH3 (black).

To investigate whether the VPP peptide contains secondary structure or highly flexible regions in the free state, we used a Random Coil Index (RCI) analysis^19^. The RCI values were calculated using the chemical shifts of ^1^Hα, ^13^Cα, ^13^C′ and ^15^N for the VPP peptide in both states. Figure 2 shows the RCI values for each residue of the VPP peptide in the free and Grb2 nSH3-bound states. In the bound state, all residues with relatively low (<0.1) RCI values represented the overall form of the VPP peptide, i.e., a secondary structure rather than a random coil. In contrast, in the free state, Pro4, Val5, and Pro6 showed low RCI values comparable to those of the bound state, whereas the other residues showed higher (>0.1) RCI values. This analysis suggests that the core (^4^PVP^6^) region of the VPP peptide forms a relatively rigid structure, even in the free state, and the N- and C-terminal (^1^VPP^3^ and^7^PRRR^10^) regions are more flexible than the core region.

### ^13^Cα relaxation dispersion analysis

Non-labelled VPP (10 mM) peptide mixed with 0.25 mM Grb2 nSH3 dissolved in ^2^H_2_O solvent was subjected to ^13^Cα relaxation dispersion experiments. Although the affinity (*K*_d_) of VPP peptide for Grb2 nSH3 has been estimated as 3.5 μM^12^, which is a relatively high affinity for a SH3 domain, a chemical exchange between the free and bound states was established by the peptide-protein mixture with a molar ratio of 40:1.

**Figure 3:**
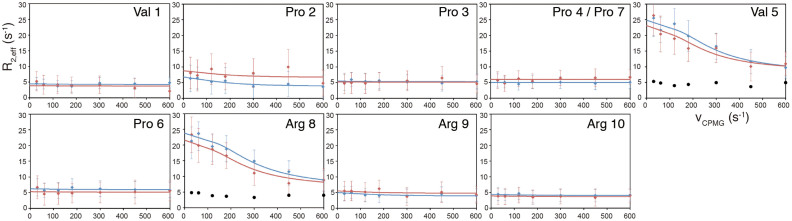
^13^Cα CPMG relaxation dispersion profiles of the VPP peptide in the presence of Grb2 nSH3 recorded under static magnetic fields of 14.1 T (blue) and 11.7 T (red). Black filled circles on Val5 and Arg8 indicate the relaxation dispersion profiles for the free state recorded at 11.7 T. Signals for Pro4 and Pro7 are overlapped at the same chemical shift.

Figure 3 shows the curves of ^13^Cα relaxation dispersion profiles for the VPP peptide recorded at static magnetic fields (11.7 and 14.1 T) and 25°C, where signals for Pro4 and Pro7 were overlapped at the same chemical shift. Significant dispersion was only observed for Val5 and Arg8 and not for the other residues. In contrast, when Grb2 nSH3 was absent, the dispersion curves were flat. Therefore, the significant dispersion curves reflect the exchange process that occurs when Grb2 nSH3 binds to the VPP peptide. The global exchange parameters of the exchange rate, *k*_ex_; the fractional population of the minor state, *P*_b_ were calculated from the dispersion curves of Val5 and Arg8. Then, the chemical shift difference between two states, Δω; the target function of fitting error, χ^2^; and the nonexchangeable contribution of R_2,eff_, R_2,0_ for each residue were calculated using the global exchange parameters based on a two-state exchange model. These results are summarized in Table 2. These parameters indicate a conformational exchange process for the VPP peptide with an exchange rate (*k*_ex_) of ~1000 s^−1^ and ~2.6% of the population in the minor state (*P*_b_). The *k*_ex_ values were slightly larger than the Δω values, indicating a fast or intermediate exchange process on the chemical shift time scale. The exchange rate *k*_ex_ is defined as *k*_ex_ = *k*_on_[P] + *k*_off_, where [P] is the concentration of the protein. In this experimental condition, [P] is assumed to be zero because the concentration of the ligand is much higher than that of the protein. Thus, *k*_ex_ = *k*_off_. The affinity of the ligand to the protein, i.e., *K*_d_, is defined as *K*_d_ = *k*_off_/*k*_on_. If *k*_on_ is diffusion-controlled, *k*_on_ ~ 10^8^ M^−1^s^−1^. Therefore, using the *k*_ex_ value, the *K*_d_ between the VPP peptide and Grb2 nSH3 is estimated to 10 ± 4 μM. The estimated *K*_d_ is similar to the experimental *K*_d_ (3.5 μM). Moreover, the value of *P*_b_ (2.6%) showed good agreement with the experimental condition of the population for the bound state (2.5%). Δω values determined by the relaxation dispersion analysis for each residue had a range of 0–1.5 ppm and roughly corresponded to the actual chemical shift differences between the two states (See Table 1 and 2). However, the Δω values for Val5 and Arg8 (1.47 and 1.53 ppm, respectively) were larger than the actual chemical shift differences between both states (0.8 and 0.6 ppm, respectively). These differences in the magnitude of the chemical shift differences were probably derived from a lack of sensitivity caused by the use of the ^13^C-nonlabelled sample. Therefore, if we used a ^13^Cα-selective labelled peptide for the present experiment, these fitting errors may have been avoided. However, as reported by Lundström et al. Cα and Cβ atoms of valine are simultaneously incorporated to the final products from [^13^C-2]-glucose as a sole carbon source by using an *E.coli* expression system^20^. Thus, isolated ^13^Cα-selective labelled valine residues cannot be prepared for this experimental purpose.

## Discussion

We investigated the conformation of the VPP peptide using the RCI method. This analysis indicated that the core region of VPP peptide takes on a somewhat rigid structure even in the free state. This result is similar to that of a recent study that used NMR and CD^14^. Moreover, we detected conformational changes at the Val5 and Arg8 residues of the VPP peptide during the transition between the free and Grb2 nSH3-bound states. Wittekind et al. have shown that Pro3, Val5, Pro6, and Arg8 of the VPP peptide are critical for interaction with Grb2 nSH3 using an isothermal titration calorimetry experiment with a single alanine substitution for the peptide^12^. Interestingly, our ^13^Cα relaxation dispersion analysis showed the conformational exchange (i.e., significantly large Δω) was not present in Pro3, Pro4/7 and Pro6. Terasawa et al. mentioned that Val5 has many intermolecular NOE relationships with Grb2 nSH3; therefore, Val5 plays a role in determining ligand specificity^10^. Arg8 also plays an important role in determining the binding orientation of the VPP peptide at the Grb2 nSH3 domain as mentioned above. The VPP peptide, which takes on a somewhat rigid structure in the free state, probably first contacts Grb2 nSH3 using Val5 and Arg8, and then the entire peptide forms a PPII-helix at Grb2 nSH3 by induced fitting. However, relaxation dispersion analyses cannot be used to evaluate residues with unchanged chemical shifts upon binding (for example, Pro3 by 0.1 ppm or Pro7 by 0 ppm). If further detail of the binding mechanism between the protein and the peptide is needed, for example, in a three-step binding analysis^21^, the dispersion data for other nuclei have to be calculated, and higher-order fitting based on multiple state models and a model-free analysis of order parameter *S*^2^ have to be performed.

In conclusion, NMR analysis based on the chemical shift and relaxation dispersion revealed that the core region of VPP peptide has a somewhat rigid conformation in the free state, and Val5 and Arg8 show a large conformational exchange upon binding to Grb2 nSH3.

## Methods

### Sample preparation

The plasmid to express the Grb2 nSH3 domain (residues 1–57) was constructed by using a pGEX-6P expression vector (GE Healthcare Life Sciences, Sweden). Transformed *E. coli* BL21 (DE3) cells were grown in LB medium at 37°C and induced at OD_600_ = 0.7 with 0.1 mM isopropyl-1-thio-β-galactopyranoside. The cells were cultured at 22°C and harvested after 12 h of induction. GST-fused Grb2 nSH3 was purified using a glutathione-Sepharose 4B column (GE Healthcare Life Sciences), and GST was excised from Grb2 nSH3 by incubation with PreScission protease (GE Healthcare Life Sciences) for 3 h at 4°C. The isolated Grb2 nSH3 was purified using a Superdex75 gel-filtration column (GE Healthcare Life Sciences) and eluted with phosphate-buffered saline (PBS).

Synthesized double-stranded DNA coding the VPP peptide derived from Sos (VPPPVPPRRR, residues 1152–1161) was cloned into a modified pET21d vector (Novagen, UK) to create a fusion protein consisting of an N-terminal His_6_-tag followed by the GB1 domain, a PreScission Protease cleavage site, and then the VPP peptide. The protein was expressed in *E. coli* BL21 Star (DE3) cells (Life Technologies, USA) grown in LB or MP media containing ^15^NH_4_Cl (2 g/l), and ^13^C-labelled glucose (4 g/l) was used for the preparation of naturally abundant or ^13^C/^15^N- labelled proteins, respectively. Cells were grown at 37°C. Protein expression was induced at OD_600_ = 0.7 by the addition of isopropyl-1-thio-β-galactopyranoside to a final concentration of 1.0 mM. The induced cells were cultured at 37°C for 3 h. All of the expressed protein was produced as insoluble inclusion bodies. The protein was solubilized and refolded using hydrostatic refolding technique^22^. Briefly, inclusion bodies containing the expressed protein were suspended in 25 mL of Tris-HCl buffer, pH 8.0 containing 250 mM NaCl, and then hydrostatic pressure was applied using M150 equipment (Barofold, USA) filled with water at room temperature with a pressure of 230 MPa for 12 h. Solubilized and refolded protein was purified using a Ni-NTA affinity resin (Qiagen, Germany). The eluted fractions were incubated for 4 h at 4°C with PreScission Protease to cleave the fusion protein. The isolated VPP peptide was purified by reverse-phase chromatography on a Resource RPC column (GE Healthcare Life Sciences) with a linear gradient of acetonitrile (1–90%) containing 0.1% trifluoroacetic acid. The recombinant VPP peptide contains five additional N-terminal residues derived from the protease cleavage site (GPLGS).

### NMR spectroscopy

The buffer system for NMR experiments was PBS in ^2^H_2_O. The NMR sample contained 1.5 mM ^13^C/^15^N-labeled VPP peptide with or without 3 mM unlabelled Grb2 nSH3 for the bound and free states, respectively. NMR measurements for backbone resonance assignments of the ^13^C/^15^N-labeled VPP peptide in the free and Grb2 nSH3-bound states were obtained on an Inova 600 MHz NMR spectrometer (Varian, USA) at 25°C using the following experiments: 2D ^1^H-^13^C HSQC, 3D HCACO, 3D HCA(CO)N, 3D HCAN, and 3D HCA(N)CO^17^. Data were processed using the NMRPipe/NMRDraw software package^23^. Peak picking and spectral analysis were performed using the Sparky program^24^. Conformational analyses based on chemical shifts were conducted using the Random Coil Index (RCI) program^19^ on the web server (http://randomcoilindex.com/).

Relaxation dispersion profiles of naturally abundant ^13^Cα of the VPP peptide in the presence of Grb2 nSH3 were recorded using a relaxation-compensated CPMG pulse sequence^25^ on Inova 600 and 500 NMR spectrometers equipped with room-temperature probeheads at 25°C. The sample contained 10 mM VPP peptide and 0.25 mM Grb2 nSH3. Two-dimensional ^1^H-^13^C correlated spectra were collected as series of data sets with CPMG frequencies (ν_CPMG_) of 30, 60, 120, 180, 300, 450, and 600 Hz. The constant-time relaxation delay T_relax_ was set to 66.7 ms. Spectra were recorded as 62 × 600 complex points with a 2.6-s repetition delay and 32 scans for each increment. The total experimental time was 24 h for both 600 and 500 MHz spectrometers.

Peak intensities of the ^13^Cα relaxation dispersion data were quantified using the Sparky program. The transverse relaxation constant, R_2,eff_, for each ν_CPMG_ frequency was calculated as R_2,eff_ = −ln(I(ν_CPMG_)/I_0_)/T_relax_, where I(ν_CPMG_) is the peak intensity obtained for a given value of ν_CPMG_ and I_0_ is the peak intensity obtained when the CPMG block is omitted. The CPMG dispersion curves were fitted numerically as a two-state exchange model using an in-house program described elsewhere^26^,^27^. The fitting calculation extracted the following terms as the global parameters: the exchange rate, *k*_ex_; the fractional population of the minor state, *P*_b_; and the residue specific parameters, including the magnitude of the chemical shift difference between two states, Δω; the target function of fitting error, χ^2^; and the nonexchangeable contribution of R_2,eff_, R_2,0_.

## Figures and Tables

**Table 1:** Chemical shifts (ppm) of the main chain atoms for the VPP peptide in the free and Grb2 nSH3-bound states

	free state			Grb2 nSH3 bound state					
Residue	^15^N	^13^Cα	^1^Hα	^13^C′	^15^N	^13^Cα	^1^Hα	^13^C′	|Δ Cα|*
V1	122.4	59.7	4.48	174.1	122.7	59.3	4.16	173.4	0.4
P2	141.5	61.5	4.71	174.1	140.6	61.0	3.57	174.0	0.5
P3	136.0	61.3	4.74	174.8	134.7	61.2	4.54	173.3	0.1
P4	134.9	62.8	4.47	176.7	135.0	62.6	4.42	176.7	0.2
V5	121.0	59.7	4.42	174.3	124.3	60.5	4.22	174.4	0.8
P6	141.2	61.5	4.70	174.8	142.6	61.4	4.84	174.3	0.1
P7	134.9	62.8	4.45	176.8	133.7	62.8	4.58	177.5	0
R8	120.6	56.0	4.32	176.2	122.1	55.4	4.15	176.0	0.6
R9	122.7	56.0	4.37	175.2	123.8	56.1	4.24	175.1	0.1
R10	127.1	57.4	4.18	180.9	128.2	57.3	4.07	180.9	0.1

*Absolute value of the ^13^Cα chemical shift difference between the free and bound states.

**Table 2:** ^13^Cα exchange parameters for VPP peptide with Grb2 nSH3

Global kinetic parameters				
*k*_ex_ = 1037.87 ± 445.09 s^−1^		*P*_b_ = 0.026 ± 0.009		
Residue specific parameters				
Residue	*R*_2,0_ (s^−1^)^a^	*R*_2,0_ (s^−1^)^b^	Δω (ppm)	χ^2^
V1	4.24 ± 0.83	3.74 ± 1.07	0.10 ± 0.15	1.13
P2	3.77 ± 1.89	6.62 ± 1.94	0.40 ± 0.26	0.81
P3	5.28 ± 1.06	5.10 ± 1.20	0.09 ± 0.18	0.39
P4/P7	5.00 ± 0.91	5.97 ± 0.89	0.00 ± 0.16	0.63
V5	7.73 ± 3.76	8.41 ± 3.63	1.47 ± 0.52	1.52
P6	5.95 ± 1.12	5.13 ± 1.25	0.10 ± 0.19	0.40
R8	6.09 ± 3.09	6.21 ± 2.89	1.53 ± 0.42	0.98
R9	3.89 ± 1.18	4.74 ± 1.13	0.23 ± 0.20	0.53
R10	4.01 ± 0.86	3.65 ± 0.92	0.13 ± 0.17	0.19

^a^at 14.1 T;

^b^at 11.7 T.
